# Precision and Agreement of Individual and Simultaneous Macular and Optic Disc Volumetric Measurements With Spectral Domain Optical Coherence Tomography

**DOI:** 10.3389/fmed.2021.764236

**Published:** 2021-11-25

**Authors:** Alberto Domínguez-Vicent, Jesper Kensén, Marika Wahlberg Ramsay, Rune Brautaset, Abinaya Priya Venkataraman

**Affiliations:** Division of Eye and Vision, Department of Clinical Neuroscience, Karolinska Institute, Stockholm, Sweden

**Keywords:** optical coherence tomography, volumetric measurement, precision, agreement, simultaneous imaging

## Abstract

**Purpose:** To evaluate the precision of individual and combined macula and optic disc volumetric analysis, and the agreement between these two scan modes with spectral domain optical coherence tomography (OCT).

**Methods:** Macular and optic disc volumetric measurements were performed with individual and combined scan protocols in one eye of 75 healthy subjects. Three repeated measurements were performed with each protocol. From the macular area, retinal thickness in nine different sectors and ganglion cell complex thickness in eight different sectors were analyzed from both scan modes. From the optic disc area, the peripapillary retinal nerve fiber layer (pRNFL) thickness in 12 clock sectors and the optic disc parameters were evaluated. For all the parameters, repeatability limit and agreement analysis were performed.

**Results:** For the retinal thickness measurements in macula, the combined scan had two to three times larger repeatability limit than the individual scan for all the sectors except the central sector, where the repeatability limit was five times larger. The limits of agreement intervals were lower than 20 μm for all sectors, except the central. The ganglion cell complex measurements also had larger repeatability limits for the combined scans, and the limits of agreement intervals were <10 μm for all sectors. For the pRNFL thickness, the repeatability values were distributed like a vertically elongated ellipse for both scans, but still the repeatability was better for individual scan compared to the combined scan. The shortest and widest interval are obtained for sectors 9 (9 μm) and 12 (40 μm), respectively. The repeatability limit was <0.15 units for all disc parameters with both scan modes.

**Conclusion:** The individual macula and optic disc scans had better repeatability than the combined scan mode, and the two scan modes cannot be used interchangeability due to the wide limits of agreement.

## Introduction

Optical coherence tomography (OCT) is an irreplaceable imaging technology that allows the acquisition of *in vivo* and non-invasive cross-sectional images of the retina and choroid (1, 2). The OCT images from the volumetric scans have been used to quantitatively evaluate the different layers of the retina, which has been proven to be valuable for the diagnosis and follow-up of various ocular diseases (3–8). Clinical available OCTs have different scanning protocols for the macular and optic disc volumetric measurements. The built-in automated segmentation algorithm provides reliable volumetric measures of different retinal layers (9–13).

Macula and optic disc areas are usually imaged separately to get the respective volumetric measurements, and the precision of these thickness measurements has been well investigated (9, 14–17). Current-generation OCTs allow wide-field visualization of the retina with a scan area that can cover both macula and optic disc (18–20). With simultaneous imaging of macula and optic disc, the measurement time can be reduced to half. In addition, the fixation target is paracentral, which can reduce the fixation errors during the image acquisition. The wide-field scan protocol has been shown to be comparable and has a diagnostic ability that is similar to that of the individual macula and optic disc scans (19, 20). In the previous studies mentioned, the thickness parameters obtained with the wide-field scanning protocol from the swept-source OCT were compared to the individual scanning protocol obtained with the spectral domain OCT. The combined scan mode is possible even with a spectral domain OCT that has a scan window that can cover both the macula and optic disc. The Canon OCT HS-100 (Canon Europe, the Netherlands), which is a spectral domain OCT, has an updated scanning protocol that performs volumetric measurements on an area of 13 ×10 mm, allowing simultaneous imaging and combined volumetric analysis of both macula and optic disc. The B-scan density for this simultaneous scan protocols is different compared to the individual scanning protocols as number of B-scans used in these scan modes are same though the area covered is different. It would be interesting to know the precision and how comparable are the thickness values obtained with individual and simultaneous scanning protocols.

The aim of the present study is to evaluate the precision of individual and combined macula and optic disc volumetric analysis, and the agreement between these two scan modes with the Canon OCT HS-100. The results of the present study help to decide whether the combined scan can substitute the individual scans in clinical practice.

## Methods

### Participants

A total of 75 healthy volunteers (28 men and 47 omen; mean age: 32.72 ± 10.28 years, range: 20–62 years) participated in this study. In order not to artificially reduce the CI around the limits of agreement (21), only one eye per participant was included (the right eye of the participants was measured if they were born in even months, and the left eye was measured if the participant was born in odd months). The study protocol adhered to the tenets of the Declaration of Helsinki and was approved by the Regional Ethical Committee (Regional ethics committee, Stockholm 2011/874-31/2). Written informed consent from all participants was obtained after explaining the purpose, nature, and the possible consequences of the study.

The inclusion criteria to participate in this study were best corrected visual acuity better or equal to 0.1 logMAR and no history of amblyopia; no history of any ocular surgery; normal anterior and posterior segment (i.e., no significant opacities, irregularities, or pathologies); and intraocular pressure below 21 mmHg.

### Instrumentation and OCT Measurements

The participants underwent OCT imaging with the Canon OCT HS-100 at the Optometry Clinic (Karolinska Institute, Solna, Sweden). The OCT HS-100 performs up to 70,000 A-scans/s with an axial resolution of 3 μm, with a maximum scan width of 13 mm. This instrument allows the possibility to image the macula and optic disc individually (with a maximum scan width of 10 and 6 mm, respectively), or simultaneously with a 13-mm scan width. Table 1 summarizes the specifications of each scan mode. The main differences between the two scan modes are the B-scan density and scan area. From this point forward, the individual image scan mode of the macula or optic disc will be referred to with the term *individual scan*, and the term *combined scan* will be used to refer to the scan mode in which the macula and optic disc are imaged simultaneously.

**Table 1:** Specifications of the individual scan and combined scan modes.

	**Individual scan**		**Combined scan**
	**Macula**	**Optic disc**	
Number of A-scan	1024	512	512
Number of B-scan	128	256	128
Scan width (mm)	10	6	13
Scan area (mm^2^)	10 x 10	6 x 6	13 x 10
B-scan orientation	Vertical	Horizontal	Vertical
Interscan distance (μm)	77	23	100

In this study, nine scans were acquired in each eye (three individual scans of the macula, three individual scans of the optic disc, and three combined scans). The repeated measurements were taken under repeatability conditions (22, 23), and with sufficient breaks in between to ensure good patient cooperation. The same experienced examiner performed all OCT scans, and these were repeated in case of poor fixation, subject blink, or signal strength <7 (out of 10).

### Parameters Analyzed

**Figure 1:**
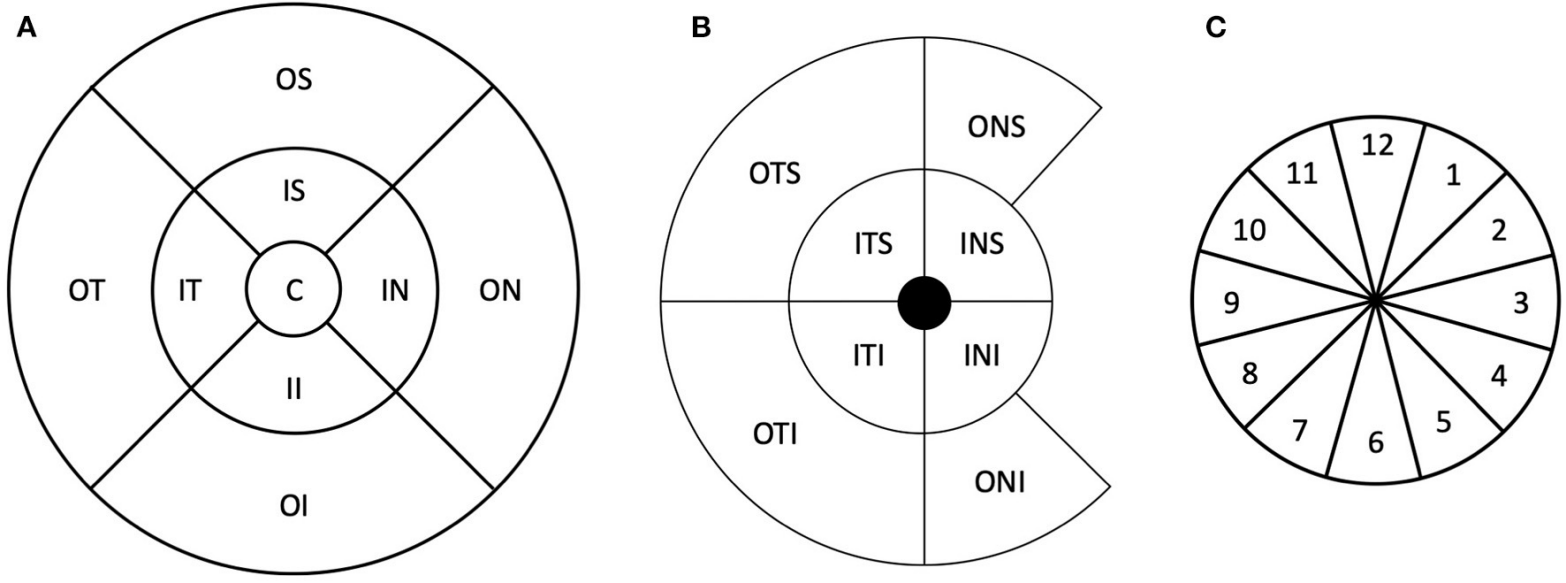
Schematic representation of the thickness maps used in this study. **(A)** Early Treatment Diabetic Retinopathy Study sector map. **(B)** Ganglion cell complex map. **(C)** Schematic representation of the peripapillary retinal nerve fiber layer thickness sectors. The numbers represent the clock hour positions. C, central; IS, inner superior; OS, outer superior; IN, inner nasal; ON, outer nasal; II, inner inferior; OI, outer inferior; IT, inner temporal; OT, outer temporal; ITS, inner temporal superior; INS, inner nasal superior; INI, inner nasal inferior; ITI, inner temporal inferior; OTS, outer temporal superior; ONS, outer nasal superior; ONI, outer nasal inferior; OTI, outer temporal inferior.

From both individual and combined scans, measurements from the macular and optic disc area were exported. From the macular area, volumetric measurements of the retinal layer thicknesses were exported. The retinal thickness was measured from the inner limiting membrane (ILM) to the retinal pigment epithelium (RPE). The Early Treatment Diabetic Retinopathy Study (ETDRS) map was used to evaluate the ILM–RPE thickness. The ETDRS map consists of a central circle, inner ring (nasal, superior, temporal, and inferior), and outer ring (nasal, superior, temporal, and inferior) with diameters of 1, 3, and 6 m, respectively. Panel A in Figure 1 shows a schematic diagram of the ETDRS map.

In addition to ILM–RPE thickness, ganglion cell complex thickness was also evaluated from retinal nerve fiber layer (RNFL), ganglion cell layer (GCL), and inner plexiform layer (IPL) thicknesses. This was exported in terms of RNFL–GCL–IPL and GCL–IPL thickness in eight sectors divided into two concentric circles with diameters of 5 and 10 mm. Panel B in Figure 1 shows the schematic representation of the ganglion cell complex map.

From the optic disc, the peripapillary RNFL (pRNFL) thickness around the optic nerve head was evaluated in 12 clock-hour sectors in a circle of 3.45 mm diameter centered at the optic disc. Panel C in Figure 1 shows a schematic representation of the clock positions. The optic nerve parameters (disc area, rim area, cup volume, and cup–disc ratio vertical and horizontal) were also obtained. All thicknesses were obtained using the automated segmentation algorithm from the OCT instrument, and no manual adjustments of the segmentation were allowed.

### Statistical Analysis

The baseline demographics of the participants and observations are summarized with descriptive statistics. The within subject SD (Sw), repeatability limits, and coefficient of variation (CoV) were used to describe the repeatability of the OCT HS-100 in both individual and combined scan modes. The Sw, which represents the repeatability of the measurements, was calculated with a one-way ANOVA. The repeatability limit was calculated as $1.96\cdot \sqrt{2}\cdot S_{w}$, and it represents the expected limits that 95% of the measurements should be within (21). The CoVs were calculated as the repeatability limit divided by the average thickness of that sector and was expressed in percentage.

The Bland–Altman test for repeated measurements was used to analyze the agreement between the individual and combined scan modes (24). All these calculations were performed for all the parameters evaluated.

## Results

### Retinal Thickness at Macula

**Figure 2:**
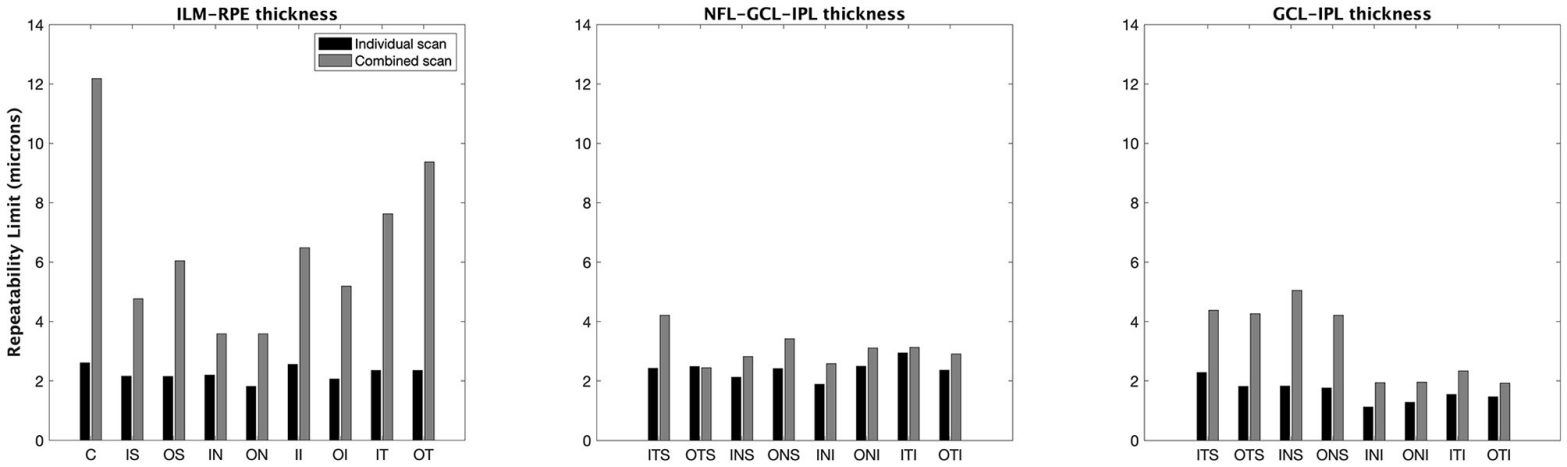
Repeatability limit for the macular thickness measurements for individual and combined scan modes. ILM–RPE, inner limiting membrane to the retinal pigment epithelium; NFL–GCL–IPL, retinal nerve fiber layer, ganglion cell layer, and inner plexiform layer; GCL–IPL, ganglion cell layer, and inner plexiform layer. See Figure 1 legend for sector abbreviations.

**Figure 3:**
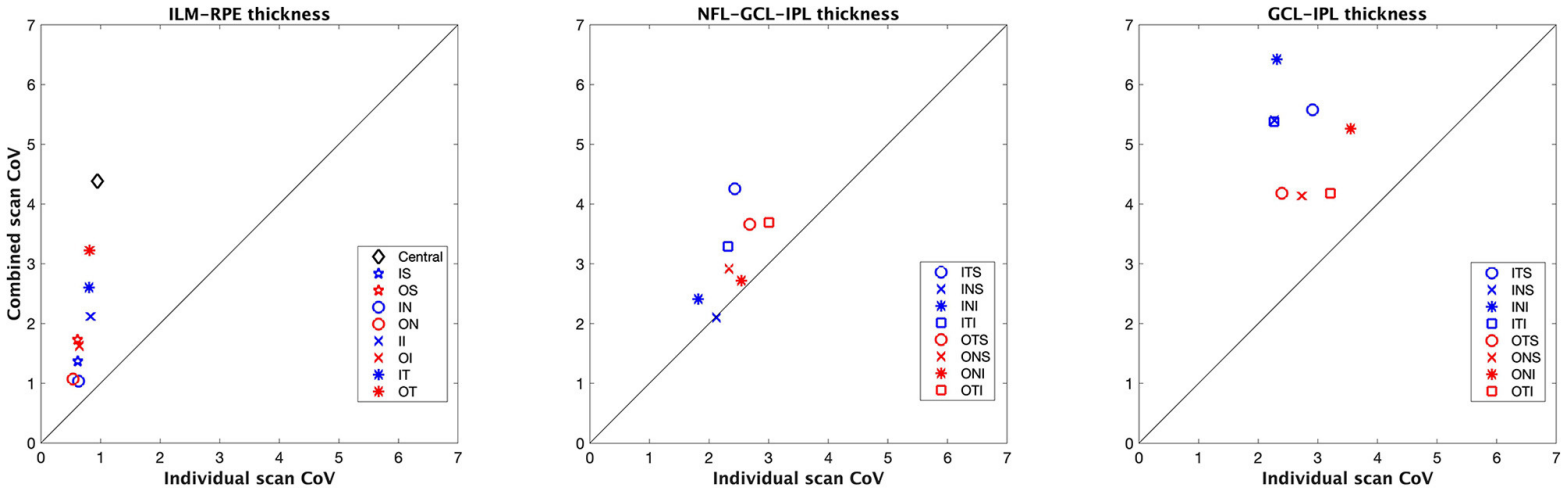
Coefficient of variation (CoV) obtained for the macular thickness measurements for individual and combined scan modes. ILM–RPE, inner limiting membrane to the retinal pigment epithelium; NFL–GCL–IPL, retinal nerve fiber layer, ganglion cell layer, and inner plexiform layer; GCL–IPL, ganglion cell layer, and inner plexiform layer. See Figure 1 legend for sector abbreviations.

Figures 2, 3 show the repeatability limit and the CoVs results, respectively, for the ILM–RPE thickness (left panel), RNFL–GCL–IPL thickness (central panel), and GCL–IPL thickness (right panel) for both scan modes. For ILM–RPE thickness, the repeatability limit values for the individual scan were about two to three times less than the combined scan for all ETDRS sectors except the central sector, where the repeatability limit for the individual scan was five times less than the combined scan. The repeatability limit values were similar among the ETDRS sectors for the individual scan, whereas the values were heterogeneous for the combined scan. Concretely, the repeatability values for the individual scan ranged from 1.80 μm (outer nasal sector) to 2.60 μm (central sector), and for the combined scan, the repeatability values ranged from 3.50 μm (outer nasal sector) to 12 μm (central sector). Regarding the CoV values for the ILM–RPE thickness, the values corresponding to all ETDRS sectors are represented on the top of the line of equality. The CoVs for the individual scan are smaller than 1% for all sectors, and the CoVs for the combined scan range from 1 to 4.5%.

Table 2 summarizes the descriptive statistics of the ILM–RPE thicknesses obtained with each scan modality, and the agreement results between the individual and combined scans for each ETDRS sector. On average, the mean difference between the individual and combined scans was lower than the axial resolution of the instrument (3 μm) for all ETDRS sectors except the central one, where the mean difference value is −3.50 μm. The limits of agreement intervals were <20 μm for all ETDRS sectors, except the central, where the interval was 22 μm.

**Table 2:** Retinal thickness in macula (Internal limiting membrane to retinal pigment epithelium) for 9 different sectors with two scan modes, and the agreement results.

**Sectors**	**Individual scan** **(mean ± STD)**	**Combined scan** **(mean ± STD)**	**Mean difference (limits of agreement)**
Central	274.47 ± 21.79	277.96 ± 23.25	−3.50 (−14.80 to 7.81)
Inner superior	349.31 ± 17.95	348.11 ± 18.01	1.20 (−3.12 to 5.53)
Outer superior	349.63 ± 17.15	349.48 ± 17.94	0.15 (−6.16 to 6.46)
Inner nasal	346.13 ± 17.24	346.28 ± 17.01	−0.15 (−5.23 to 4.94)
Outer nasal	335.90 ± 16.02	335.52 ± 15.99	0.38 (−3.68 to 4.45)
Inner inferior	304.93 ± 16.98	305.74 ± 17.43	−0.81 (−7.67 to 6.10)
Outer inferior	319.84 ± 21.40	319.93 ± 21.99	−0.10 (−5.10 to 4.91)
Inner temporal	290.86 ± 16.86	293.00 ± 16.68	−2.14 (−10.96 to 6.68)
Outer temporal	288.33 ± 15.96	290.81 ± 15.52	−2.48 (−11.64 to 6.67)

*All values are expressed in microns*.

*STD, standard deviation*.

### RNFL–GCL–IPL Thickness at Macula

The repeatability limit for the individual scan mode was <3 μm in all eight sectors (Figure 2, central panel). This scan mode had lower repeatability values compared to the combined scan for all sectors, except one, the inner nasal superior where the values were similar. The repeatability limit values were homogeneous among all sectors with both scan modes. Concretely, the outcomes ranged from 1.9 to 2.9 μm for the short scan and from 2.4 to 4.2 μm for the large scan.

From the CoV results (Figure 3, central panel), the values corresponding to the combined scan were larger than those from the individual scan for all sectors except for the inner nasal superior sector, where the CoVs from both scan modes were the same. The CoVs obtained with the individual and combined scans ranged from 2 to 3%, and 2.5 to 4 %, respectively.

The descriptive statistics of the NFL–GCL–IPL and GCL–IPL thicknesses obtained with each scan modality, and the agreement results between both scan modes for each sector are included in Table 3. On average, the mean difference between the individual and combined scan modes was <1 μm for all sectors. The limits of agreement intervals were similar among all sectors, the minimum and maximum intervals being 5 and 8 μm, respectively.

**Table 3:** Ganglion cell complex thickness in macula for 8 different sectors with two scan modes, and the agreement results.

	**NFL-GCL-IPL**			**GCL-IPL**		
**Sectors**	**Individual scan**	**Combined scan**	**Mean difference**	**Individual scan**	**Combined scan**	**Mean difference**
	**Mean ± STD**	**Mean ± STD**	**(limits of agreement)**	**Mean ± STD**	**Mean ± STD**	**(limits of agreement)**
Inner temporal superior	99.49 ± 7.50	99.08 ± 7.54	0.41 (−3.81 to 4.62)	78.42 ± 6.77	78.50 ± 6.81	−0.08 (−4.32 to 4.16)
Outer temporal superior	116.89 ± 9.00	116.18 ± 9.22	0.71 (−2.02 to 3.45)	79.95 ± 7.32	79.04 ± 8.01	0.91 (−3.90 to 5.72)
Inner nasal superior	117.09 ± 9.91	116.86 ± 10.02	0.23 (−2.62 to 3.07)	79.03 ± 8.40	78.64 ± 8.98	0.39 (−4.50 to 5.27)
Outer nasal superior	104.09 ± 7.93	103.89 ± 7.63	0.20 (−3.65 to 4.05)	77.75 ± 6.64	78.36 ± 6.56	−0.62 (−5.26 to 4.03)
Inner nasal inferior	70.54 ± 5.49	70.63 ± 5.31	−0.09 (−3.05 to 2.87)	46.83 ± 3.80	46.54 ± 3.84	0.29 (−1.56 to 2.14)
Outer nasal inferior	106.90 ± 10.46	106.66 ± 10.07	0.24 (−3.52 to 4.00)	46.80 ± 3.82	47.44 ± 3.92	−0.64 (−2.79 to 1.50)
Inner temporal inferior	115.89 ± 11.66	115.00 ± 11.59	0.90 (−2.60 to 4.40)	43.49 ± 4.30	44.42 ± 4.56	−0.92 (−3.33 to 1.48)
Outer temporal inferior	78.65 ± 6.94	78.77 ± 6.67	−0.12 (−3.17 to 2.93)	45.62 ± 3.84	46.13 ± 3.81	−0.52 (−2.72 to 1.69)

*All values are expressed in microns. NFL, retinal nerve fiber layer; GCL, ganglion cell layer; IPL, inner plexiform layer; STD, standard deviation*.

### GCL–IPL Thickness at Macula

The repeatability limit values obtained with the individual scan mode were 1.5 to 2.0 times lower than those obtained with the combined scan for all sectors (Figure 2, right panel). With the individual scan mode, the repeatability limit was similar among all sectors, and it was in all cases lower than 3 μm. The repeatability limit for the combined scan mode was different among the sectors. Concretely, the repeatability values for the outer sectors were two times lower than their corresponding inner sectors.

The combined scan had larger CoV values compared to the individual scan for the GCL–IPL thickness in all sectors (Figure 3, right panel). In addition, the outer sectors had lower CoV values compared to their respective inner sectors.

The mean difference between both scan modes was <1 μm for all sectors (Table 3). The limits of agreement intervals were <10 μm for all sectors. However, the values for the inner sectors were twice than that of the corresponding outer sectors.

### PRNFL Thickness

**Figure 4:**
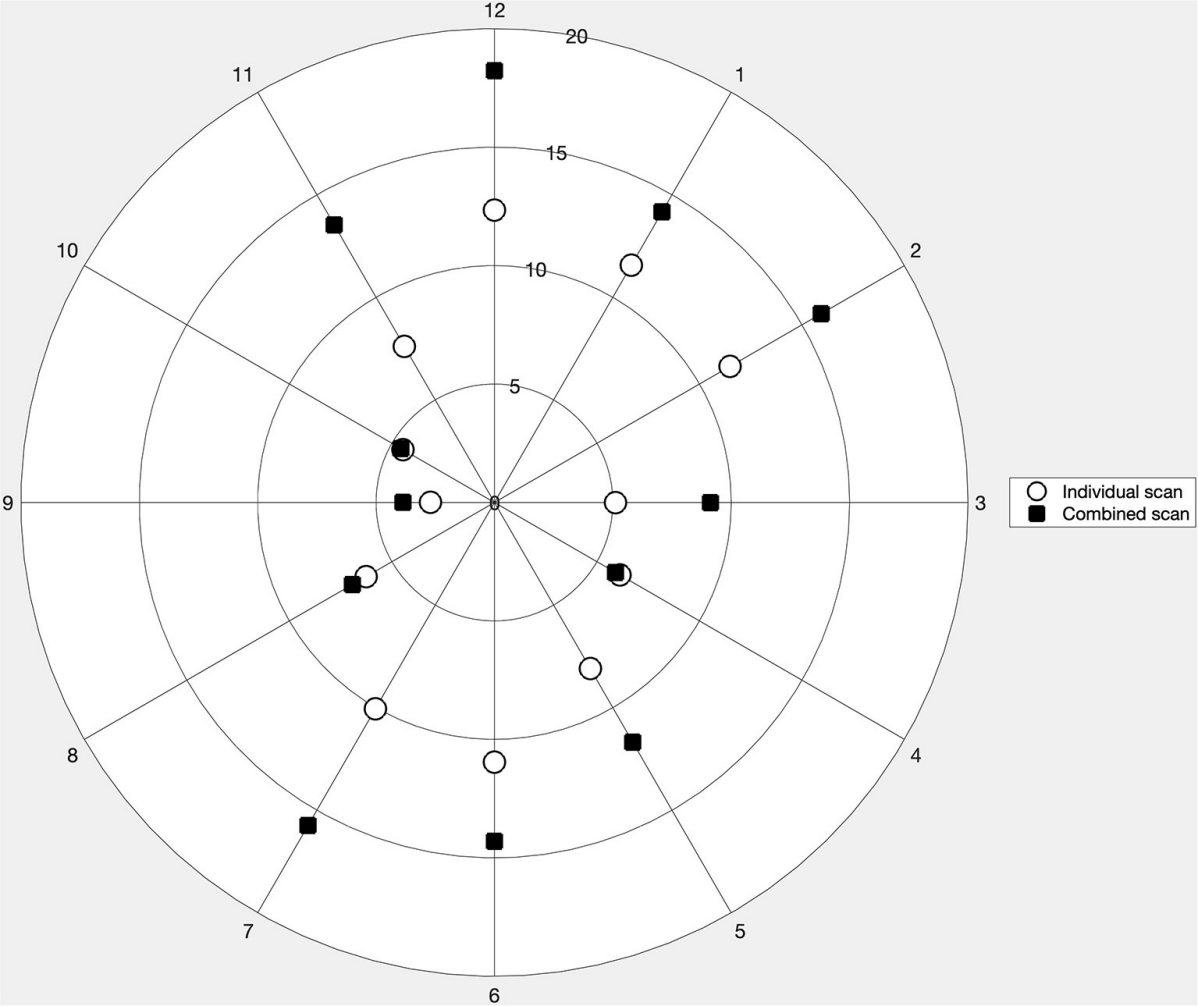
Repeatability limit for the peripapillary retinal nerve fiber layer thickness measurements for the 12 clock sectors obtained with the individual and combined scan modes represented in a polar plot.

The repeatability of the three consecutive measurements of pRNFL thickness for the individual and combined scan modes is represented in a polar plot (Figure 4). In both scan modes, the repeatability limit never exceeded 20 μm in any of the sectors. The repeatability limit for the individual scan was lower than that of the combined scan for all clock positions except for clock positions 4 and 10, where the values were almost the same. It can also be noticed that the values for the individual and combined scan are distributed like a vertically elongated ellipse. Sector 9 showed the best repeatability with both scan modes, with the repeatability value of 2.7 and 3.8 μm, respectively. Both scan modes showed the worst repeatability in sector 12, with a repeatability value of 12.30 μm for the individual scan and 18.22 μm for the combined scan.

Table 4 summarizes the descriptive statistics of the pRNFL thicknesses obtained with each scan modality, and the CoV and agreement results between the individual and combined scans for each clock sector position. The combined scan had larger CoV values compared to the individual scan in all clock sectors. The mean difference between both scan modalities never exceeded 7 μm in any of the clock position. Nonetheless, the limits of agreement interval were lessr than 20 μm for sectors 3, 4, 9, and 10, and larger than 20 μm for the other sectors. The shortest and widest intervals are obtained for sectors 9 (9 μm) and 12 (40 μm), respectively.

**Table 4:** Peripapillary nerve fiber layer thickness for 12 clock sector position with two scan modes, and the coefficient of variation (CoV), and the agreement results.

	**Individual scan**		**Combined scan**		**Mean difference (limits of agreement) (Values in microns)**
	**Mean ± STD (in microns)**	**CoV (%)**	**Mean ± STD (in microns)**	**CoV (%)**	
Clock position 1	115.69 ± 24.27	4	114.98 ± 22.92	12	0.71 (−15.05 to 16.48)
Clock position 2	107.37 ± 20.97	4	110.12 ± 21.50	14	−2.75 (−18.74 to 13.23)
Clock position 3	71.19 ± 15.10	2	73.15 ± 16.20	12	−1.96 (−10.89 to 6.96)
Clock position 4	71.62 ± 14.57	2	70.79 ± 14.58	8	0.83 (−6.84 to 8.51)
Clock position 5	108.20 ± 21.66	3	105.26 ± 21.29	11	2.94 (−9.32 to 15.21)
Clock position 6	138.07 ± 26.33	4	134.20 ± 26.44	11	3.87 (−12.42 to 20.16)
Clock position 7	152.67 ± 20.19	3	157.59 ± 21.50	10	−4.92 (−23.30 to 13.47)
Clock position 8	81.29 ± 14.42	2	87.84 ± 15.30	8	−6.54 (−17.34 to 4.26)
Clock position 9	55.67 ± 6.75	1	58.25 ± 7.05	7	−2.58 (−6.85 to 1.68)
Clock position 10	78.80 ± 12.04	2	80.25 ± 12.11	6	−1.44 (−7.32 to 4.43)
Clock position 11	127.44 ± 21.94	3	130.66 ± 22.53	10	−3.23 (−17.78 to 11.32)
Clock position 12	133.56 ± 20.73	4	131.48 ± 22.58	14	2.08 (−18.18 to 22.35)

*STD, standard deviation*.

### Optic Disc Parameters

The descriptive statistics, repeatability, and agreement results for the optic nerve parameters obtained with the individual and combined scan modes are summarized in Table 5. The repeatability limit was <0.15 unit for all parameters independently of the scan mode. Nevertheless, the repeatability values obtained with the individual scan were lower than those obtained with the combined scan. Concretely, these values were about two to three times lower. The CoVs for disc and rim area were also lower in the individual scan mode.

**Table 5:** Optic nerve parameters with two scan modes, and the repeatability, and agreement results.

**Optic nerve parameter**	**Individual scan**			**Combined scan**			**Mean difference** **(limits of agreement)**
	**Mean ± STD**	**Repeatability limit**	**CoV (%)**	**Mean ± STD**	**Repeatability limit**	**CoV (%)**	
Disc area (mm^2^)	2.05 ± 0.35	0.05	2%	2.06 ± 0.35	0.14	7%	−0.01 (−0.12 to 0.11)
Rim area (mm^2^)	1.41 ± 0.49	0.04	3%	1.32 ± 0.55	0.10	7%	0.09 (−0.64 to 0.82)
Cup volume (mm^3^)	0.11 ± 0.12	0.04	N/A	0.11 ± 0.12	0.07	N/A	−0.01 (−0.07 to 0.06)
Cup-disc ratio vertical	0.44 ± 0.19	0.03	N/A	0.44 ± 0.20	0.04	N/A	0.00 (−0.06 to 0.05)
Cup-disc ratio horizontal	0.48 ± 0.21	0.03	N/A	0.48 ± 0.22	0.06	N/A	0.00 (−0.07 to 0.07)

*CoV, coefficient of variation*.

*N/A, values not applicable due the high standard deviation*.

Regarding the agreement, the mean difference between the individual and combined scans was close to zero for all optic nerve parameters. The limits of agreement interval were narrower than 0.15 units for all parameters, except the Rim area, where the agreement interval was 1.50 mm^2^.

## Discussion

The precision of individual and combined macula and optic disc OCT scans, and their agreement were evaluated. In general, the individual scan had a better repeatability than the combined scan for all thickness measurements. The mean difference in the thickness measurements between the two scan modes was good in general except for the pRNFL thickness in some of the sectors.

In the macular area, there is a notable difference in the repeatability limit between the scan modes for the ILM–RPE thickness. For the ganglion cell complex thickness, though the individual scan had better repeatability than the combined scan, the difference is not as large as in the ILM–RPE thickness. Both the repeatability and CoV values were more homogenous among the sectors for the individual scan mode compared to the combined scan mode. The agreement between the two scan modes were not uniform among the sectors. Though the number of B-scans is the same for both scan modes, the combined scan covers a larger area than the individual scan. This results in a larger interscan distance for the combined scan, which could explain the worse repeatability. It has been reported that the reduction in the B-scan density is associated with an increase in the error of the retinal thickness measurement (25). Larger agreement intervals were seen in those sectors that had bad repeatability with the wide scan mode. This is not unexpected as low repeatability in one or both measurement modes is known to result in low agreement (24). Similarly, a previous study has reported the worst agreement for ILM–RPE thickness in the central sector (26).

The repeatability of pRNFL measurements was heterogeneous among the sectors with worse values in the vertical sectors for both the individual and combined scans. In two of the horizontal sectors, the repeatability values were almost the same for both scan modes. In all other sectors, the repeatability of the individual scan was better than combined scan. It has been reported previously that the scan direction affects the precision of the pRNFL measurements (12), where the horizontal sectors had better repeatability with horizontal scanning, whereas with the vertical scanning the repeatability was more homogeneous. In the present study, the combined scan, which had vertical B-scans, did not have a homogeneous repeatability among the sectors. The scan density in the optic disc for the combined scan is 4.4 times less than for the individual scan. This can explain the differences in the repeatability values between the scan modes and among the sectors. Improving the B-scan density in the combined scan mode could improve the repeatability though that might increase the acquisition time. The agreement between both scan modes for the pRNFL measurements was worse in the sectors that had low repeatability. Ideally, the comparison between individual and combined scan modes would be more meaningful if the scan direction would have been the same. However, the B-scan directions are fixed for both scan modes and cannot be modified. The repeatability of all the optic disc parameters was good with both scan modes. The agreement was also good for all parameters except the rim area. These results on the optic nerve parameters are similar to those obtained in previous studies (15, 16, 27).

The diagnostic ability of the wide scanning protocol for early glaucoma diagnosis has been studied previously (19, 20), and it has been reported that the glaucoma-discriminating ability of the combined scan was comparable to that of the individual macula and optic disc scans. In the present study, we evaluated the precision and agreement of the individual and combined scan modes in healthy eyes, and it would be interesting to assess these metrics in eyes with glaucoma. Based on the current results, it can already be suggested that the same scan mode should be used for follow-up measurements as we can expect the same tendency in eyes with glaucoma also.

The minimum number of measurements (*N*) needed to ensure a certain measurement tolerance can be estimated as $\frac{1.96\;\cdot S_{w}}{\sqrt{N}}$ (22, 23). Based on the current repeatability results, to achieve a measurement tolerance of 6 μm (twice the instrument's axial resolution), the individual scan protocol requires only one volumetric scan for the macular thickness measurements, and two volumetric scans for the pRNFL measurements in healthy eyes. With the combined scan protocol, two and five volumetric scans are needed for the macular and pRNFL measurements in healthy eyes, respectively, to achieve a measurement tolerance of 6 μm.

In conclusion, the individual macula and optic disc scans had better repeatability than the combined scan mode, and the two scan modes cannot be used interchangeability due to the wide limits of agreement.

## Data Availability Statement

The original contributions presented in the study are included in the article/supplementary material, further inquiries can be directed to the corresponding author.

## Ethics Statement

The studies involving human participants were reviewed and approved by Regional Ethics Committee, Stockholm. The patients/participants provided their written informed consent to participate in this study.

## Author Contributions

AD-V and AV: conception and design of the work, analysis and interpretation of data, drafting, and revising the manuscript. JK: conception and design, acquisition, and revising the manuscript. MR and RB: conception and design of the work, interpretation of data, and revising the manuscript. All authors provide approval for publication of this manuscript and agree to be accountable for all aspects of the work.

## Funding

AD-V: 2021—promoting vision research fund, Ögonfonden, Sweden. AV: 2020—promoting vision research fund, Ögonfonden, Sweden. The funders had no role in study design, data collection and analysis, decision to publish, or preparation of the manuscript.

## Conflict of Interest

The authors declare that the research was conducted in the absence of any commercial or financial relationships that could be construed as a potential conflict of interest.

## Publisher's Note

All claims expressed in this article are solely those of the authors and do not necessarily represent those of their affiliated organizations, or those of the publisher, the editors and the reviewers. Any product that may be evaluated in this article, or claim that may be made by its manufacturer, is not guaranteed or endorsed by the publisher.
